# Assessing the association of single nucleotide polymorphisms in thyroglobulin gene with age of puberty in bulls

**DOI:** 10.1186/2055-0391-56-17

**Published:** 2014-09-16

**Authors:** María Elena Fernández, Daniel Estanislao Goszczynski, Alberto José Prando, Pilar Peral-García, Andrés Baldo, Guillermo Giovambattista, Juan Pedro Liron

**Affiliations:** Instituto de Genética Veterinaria (IGEVET), CCT La Plata – CONICET - Facultad de Ciencias Veterinarias, Universidad Nacional de La Plata, Calle 60 y 118 s/n, La Plata, B1900AVW, CC 296 Argentina; Departamento de Producción Animal, Facultad de Ciencias Veterinarias, Universidad Nacional de La Plata, La Plata, Argentina; Fellow of the Consejo Nacional de Investigaciones Científicas y Técnicas (CONICET), La Plata, Argentina

**Keywords:** Bovine, Puberty, Polymorphism, Thyroglobulin, Association study

## Abstract

Puberty is a stage of sexual development determined by the interaction of many loci and environmental factors. Identification of genes contributing to genetic variation in this character can assist with selection for early pubertal bulls, improving genetic progress in livestock breeding. Thyroid hormones play an important role in sexual development and spermatogenic function. The objective of this study was to evaluate the association between single nucleotide polymorphisms (SNPs) located in thyroglobulin(TG) gene with age of puberty in Angus bulls. Four SNPs were genotyped in 273 animals using SEQUENOM technology and the association between markers and puberty age was analyzed. Results showed a significant association (P < 0.05) between these markers and puberty age estimated at a sperm concentration of 50 million and a progressive motility of 10%. This is the first report of an association of TG polymorphisms with age of puberty in bulls, and results suggest the importance of thyroidal regulation in bovine sexual development and arrival to puberty.

## Background

Puberty is a stage of sexual development determined by the interaction of many loci hierarchically arranged in networks, and environmental factors (Ojeda et al., [1]). Puberty in cattle is an important target for genetic improvement so early prediction using genetic markers is a goal for livestock breeding. The identification of new genes and/or mutations contributing to genetic variation in puberty can assist with the selection for early pubertal bulls, reducing the generation interval and increasing fertility and genetic progress (Johnston et al., [2], Fortes et al., [3]).

Thyroid hormones (THs) exert a broad range of effects on metabolism, growth, homeostasis control, and other biological processes (Warner and Mittag, [4], Mullur et al., [5]), and show connection with nearly every biological endocrine system. Several studies provide evidence to confirm the role of THs in sexual differentiation and gonadal development (Jannini et al., [6], Mendis-Handagama and Siril Ariyaratne, [7], Flood et al., [8], Duarte-Guterman et al., [9]). For example, it has been discovered that deiodinases [the enzymes responsible for ioding thyroglobulin (TG) to obtain the active forms triiodothyronine (T3) and thyroxine (T4)] and thyroid receptors (TRs, encoded by *trα* and *trβ* genes) are present within gonadal tissues, suggesting that THs must have an action on these organs (Wagner et al., [10]). The presence of TH machinery in testicular tissues implies that TH axis must regulate aspects of testicular functioning. Indeed, it has been shown that hypo- and hyperthyroid males exhibit testes and sperm dysfunction (Krassas et. al., [11]). In addition, THs are involved in the regulation of androgen receptors(ARs) expression in testicular tissues through thyroid response elements (TREs) located in the promoter of *ar* genes(Flood et al., [8]). Furthermore, THs may also regulate other genes involved in androgen biosynthesis and signaling. For example, THs enhance 5α-reductase expression and activity within the testes, increasing 5α-dihydrotestosterone concentrations (Ram and Waxman, [12], Duarte-Guterman et al., [9]). On the other hand, it has been reported that TH-related transcription factors influence the expression of *sox9,* which induces differentiation of the bipotential cells in the testes into Sertoli cells (Zhou et al., [13]). S*ox9* stimulates the nuclear receptor steroidogenic factor 1 (*sf1*), which is primarily expressed in Leydig cells and plays an important role in sexual differentiation (Zhao et al., [14]). *Dax1* gene works in parallel with *sf1*to regulate testicular differentiation and can regulate TH-related gene expression (Sugawara et. al., [15]).

Beyond the fact that THs have considerable effects on the hypothalamic-pituitary-gonadal axis (HPG) and sexual development, some studies examined the involvement of androgens in TH synthesis and metabolism. For example, gonadotropin-releasing hormone (GnRH) interferes with the hypothalamic-pituitary-thyroid axis (HPT), increasing thyroid stimulating hormone (TSH) secretion. Moreover, ARs were identified in the thyroid gland of different vertebrate species, suggesting that the androgen axis directly regulates TH synthesis and metabolism (Pelletier et al., [16]). Other works have demonstrated the potential of ARs to regulate TH axis showing that TRs transcriptional levels and distribution within testes are responsive to androgen fluctuations through the presence of androgen response elements (AREs) in the promoter regions of TH-related genes. These evidences demonstrate the existence of a considerable cross-regulation between both axes.

Considering the existing evidence on the biological connection between metabolic status, regulated to a large extent by thyroid hormones, and sexual development, and taking into account the fact that THs play a role in the regulation of sexual function, we decided to evaluate the possible associations between SNPs located in the 3’ flanking region of TG gene with the age of puberty in bull calves.

## Methods

In order to reach this objective, DNA was extracted from blood samples belonging to 273 Angus bulls using Wizard Genomic kit, following manufacturer instructions (Promega, Madison, WI, USA). Four SNPs (rs378215592, rs110406764, rs109662686, rs109057985) in TG previously reported by Hou et al., [17] were genotyped using SEQUENOM platform by GeneSeek Inc. genotyping services (Lincoln, NE, USA). This technology is based on primer-extension reaction that generates allele-specific products with distinct masses, which are then detected through MALDI-TOF mass spectrometry (http://www.sequenom.com/). Detailed information of the studied SNPs is presented in Table 1. Animal samples, phenotypic measurements and estimation of puberty ages used in this work were reported in Liron et al., [18]. The estimated puberty ages were: i. age at 28 cm of scrotal circumference (SC28), and ii. age at sperm concentration 50 million and percentage of progressive motility 10% (C50 - M10). In order to evaluate the linkage disequilibrium (LD) between the three studied SNPs, the haplotypes for each individual were constructed using Phase algorithm (Li and Stephens, [19]).

**Table 1:** **Information of single nucleotide polymorphisms (SNP) used in this work**

SNP	Gene-Chromosome	Gene region	Position (UMD 3.1)	Allele change
rs378215592	TG- Ch14	3′ UTR	9281431	T/C
rs110406764	TG- Ch14	3′ UTR	9281469	G/A
rs109662686	TG- Ch14	3′ UTR	9281507	A/G
rs109057985	TG- Ch14	3′ UTR	9281510	T/G

The association between haplotype markers of TG gene and the estimated puberty ages was analyzed utilizing MIXED procedure implemented in SAS 9.0 software (SAS Inst. Inc.). The linear mixed model used to analyze the association between puberty age and genotypes was the following:


Where Y_ijkl_ = phenotypic observation of the I bull, μ = the overall mean, S_i_ = the fixed effect of i^th^ year, G_j_ = the fixed effect of j^th^ genotype, B_k_ = the fixed effect of k^th^ herd, O_l_ = random effect of l^th^ sire, and e_ijkI_ = random error.

## Results and discussion

After analyzing the genotyping results, one SNP (rs109662686) was removed given that it exhibited a call rate lower than 57%. The LD analysis indicated that the three remaining SNPs were completely linked (r^2^ = 1). Only two (TGT and CAG) of the eight possible haplotypes were found, with gene frequencies of 0.81and 0.19 for TGT and CAG haplotypes, respectively. Genotype frequencies values were 0.64 for homozygote TGT, 0.03 for homozygote CAG and 0.33 for heterozygote bulls. The obtained haplotypes for the 273 bulls were tested for association with phenotypic data for the two estimated puberty ages mentioned above. The association analysis showed a significant association (P < 0.05) between the haplotype markers and puberty age estimated at C50 and M10. Homozygote TGT exhibited a mean +/- S.E. age at C50 and M10 of 289.74 ± 8.13 days, while homozygote CAG showed 347.67 ± 22.51 days, resulting in a difference of 57.93 days of age. Heterozygote animals showed a mean +/- S.E. age of 299.65 ± 8.72 days of age. No significant association was found between both haplotypes and age at puberty estimated at SC28 (TGT/TGT = 277.95 ± 23.85 days, TGT/CAG = 281.09 ± 25.82 days and CAG/CAG = 292.00 ± 17.76 days, P > 0.05).

The results obtained here constitute the first report of an association of TG gene polymorphisms with age of puberty in bulls and could be explained by the vast amount of works in which the modulatory influence of THs on male reproduction is demonstrated (Flood et al., [8], Duarte-Guterman et al., [9]). The potential of THs in the modulation of male reproductive functions during or preceding puberty was determined to be of such importance at the point that any alteration in their expression and/or concentration has profound effects on male reproduction (Krassas et al., [11], Weber et al., [20]). Despite the indirect regulation of sexual maturation by THs through their known roles in development, metabolism, hormonal regulation and other physiological processes, evidence indicates that THs have direct effects on sexual development, reproductive function and associated molecular mechanisms and pathways. Although the specific mechanisms underlying this regulation are not completely established, these direct effects could be exerted mainly through the presence of TH machinery in gonadal tissues. For example, TRs are widely distributed and expressed in different compartments of the testis in mammalian species, which suggests a direct regulatory role for THs in male gonadal development and function (Jannini et al., [6], Kumar et al., [21], Wagner et al., [10]). Studies have also identified deiodinases in the testes of vertebrate species, whose role within testicular functioning in mammalian species has been reviewed by Wagner et al., [10]. Another evidence for the involvement of THs in puberty is the presence of TREs in androgen receptors in the testis and in GnRH and luteinizing hormone receptor (LHR) promoter region (Tsai-Morris et al., [22]), which demonstrates that THs can directly regulates androgen biosynthesis. Hyper- and hypo-thyroidic conditions alter GnRH concentrations in mammals, consequently affecting LH and FSH production and secretion (Chiao et al., [23]). Furthermore, several studies have shown a fall in circulating testosterone levels in hypothyroid humans (Kumar et al, [21]). As we said before, this regulation is bidirectional and there area lot of studies describing the considerable cross-regulation existing between HPT and HPG axes in vertebrates. Interestingly, Fortes et al., [24] detected SERPINA7 gene on chromosome X associated with percentage of normal sperm (PNS) in Brahman bulls. This gene codes for thyroxine-binding globulin (TBG), the major TH transport protein in serum. This evidence reinforces the hypothesis that THs play an important role in bovine male fertility.

Despite the broad range of effects in vertebrates and the connections in nearly every biological endocrine system, a lot of studies provide enough evidence to confirm the role of THs in sexual differentiation and gonadal development in mammalian and non-mammalian species. We can affirm that THs influence steroidogenesis and spermatogenesis and as described above there is extensive evidence that links TG gene to testicular development, existing a direct crosstalk between HPG and thyroid hormones axis (Wagner et al., [10], Nonneman et al., [25]).

## Conclusions

In conclusion, we detected an association between TG polymorphisms and age at puberty at C50 and M10 in male Angus cattle. Our results could contribute to the investigation on regulation of bovine puberty and fertility. THs are essential for normal growth, sexual development and reproductive function, and normal thyroid activity seems to be a requisite for an adequate male reproductive function. However, the knowledge about the interaction between both endocrine axis, HTP and HTG, is still rudimentary and needs further investigation.
